# The impact of oral health literacy on oral health in lung cancer patients: the mediating role of psychosocial factors

**DOI:** 10.3389/fpubh.2026.1890130

**Published:** 2026-09-03

**Authors:** Nan Zhang, Yingao Liu, Tiane Fa

**Affiliations:** 1 Nursing Department, Tianjin Chest Hospital, Tianjin, China; 2 Cardiology Department, Tianjin Chest Hospital, Tianjin, China

**Keywords:** anxiety, family support, lung cancer, mediating effect, oral health literacy

## Abstract

**Background:**

Lung cancer patients often have poor oral health due to treatment toxicities, increasing risks of oral mucositis, and pulmonary infection. Oral health literacy (OHL) is a key determinant, and psychosocial factors such as anxiety and family support also play roles. However, prior studies examined only single dimensions, not underlying mechanisms. This study aims to build a mediation model linking OHL, psychosocial factors, and oral health status in lung cancer patients, providing exploratory evidence for potential targets (i.e., OHL and psychosocial factors) that may warrant attention during antitumor therapy.

**Methods:**

This study recruited 250 lung cancer patients from a tertiary hospital in China to participate in a questionnaire survey. The study was conducted on a voluntary, anonymous basis, assessing patients’ general demographic information, relevant risk factor scales, the HeLD-14, oral health (BOAS, HBS, and OHIP-14), and psychosocial factors (PSS-Fa, SAS, and C-SUPPH). Finally, structural equation modeling was used to explore the interaction pathways among these three factors.

**Results:**

The oral health status score for lung cancer patients was (9.14 ± 1.94) points, the oral health literacy score was (28.07 ± 5.39) points. Notably, oral health status among lung cancer patients was significantly positively correlated with both oral health literacy (*r* = 0.389, *p* < 0.001) and psychosocial factors (*r* = 0.402, *p* < 0.001). Furthermore, psychosocial factors were significantly positively correlated with oral health literacy (*r* = 0.333, *p* < 0.001). Importantly, psychosocial factors partially mediated the relationship between oral health literacy and oral health status in lung cancer patients, with an indirect effect size of 0.264 (95% *CI*: 0.106–0.351), accounting for 49.70% of the total effect.

**Conclusion:**

Psychosocial factors partially mediate the relationship between oral health literacy and oral health status. These findings suggest a potential pathway linking OHL to oral health status through psychosocial factors. From a clinical perspective, addressing both OHL and psychosocial factors may be a reasonable strategy to support oral health in lung cancer patients, although the cross-sectional design warrants cautious interpretation. Prospective studies are needed to confirm these relationships before firm clinical recommendations can be made.

## 1 Introduction

According to 2022 cancer statistics (1, 2), lung cancer ranks first globally in terms of both incidence and mortality among malignant tumors. In China, new cases of lung cancer account for 21.98% of all malignant tumors, and deaths account for 28.49%, making it one of the most serious public health challenges (2). Changes in the tumor microenvironment caused by anticancer therapy, along with treatment-related toxicities, significantly increase the risk of complications such as oral mucositis in lung cancer patients, posing a serious threat to their oral health (3). Due to the anatomical connection between the oral cavity and the lower respiratory tract, as well as the ability of microorganisms to migrate, an imbalance in oral health can directly trigger pulmonary infections, adversely affecting patient prognosis (4–6).

Oral health status is influenced by multiple factors, among which oral health literacy (OHL) is a key determinant of an individual’s oral health status (7) Oral health literacy refers to an individual’s ability to access, process, and understand basic oral health information and services, and to make health-promoting decisions based on the resources obtained (8). Studies have found that psychosocial factors such as anxiety (9) and family support (10) significantly influence patients’ oral health status. In lung cancer patients, psychosocial factors have been empirically demonstrated to significantly influence disease management and health outcomes. A structural equation model study conducted in Chongqing found that health literacy, self-efficacy, and social support were significantly correlated with quality of life in lung cancer patients (11). Among young and middle-aged patients undergoing lung cancer surgery, health literacy and control efficacy significantly mediated the relationship between perceived social support and health-promoting lifestyles (12). Yang et al. reported that among 220 advanced lung cancer patients, higher health literacy was indirectly associated with lower fear of progression through the mediation of better symptom experience, and family support was directly correlated with lower fear of progression (13). These findings collectively indicate that in the lung cancer population, psychosocial factors—particularly anxiety, family support, and self-efficacy—are significantly associated with health literacy and health outcomes, providing an empirical basis for examining the mediating pathways through which OHL may influence oral health via psychosocial mechanisms.

In cancer patient populations beyond lung cancer, the association between oral health literacy and oral health outcomes through psychosocial pathways has received increasing empirical attention. Li et al. demonstrated that oral health literacy is associated with oral health-related quality of life through the mediating effect of oral health-related self-efficacy among inpatients (14). Similarly, all dimensions of oral health literacy have been found to correlate with oral health-related quality of life in patients with bladder cancer (15). These findings provide direct empirical support for the hypothesized mediation pathway—that oral health literacy may influence oral health outcomes through psychosocial mechanisms—and suggest that this pathway operates across different patient populations.

However, existing studies have predominantly focused on head and neck, oral, or bladder cancer patients; lung cancer—the most prevalent malignancy worldwide—remains notably understudied in this regard, despite the unique oral health risks posed by anticancer therapies and the anatomical vulnerability linking oral cavity health to pulmonary complications. Although individual associations between OHL, psychosocial factors, and health outcomes have been reported in lung cancer patients, the integrated mediating pathway of OHL → psychosocial factors → oral health status has not been systematically examined in this population.

The present study was guided by the statistical association patterns Linking Health Literacy to Health Outcomes Model proposed by Paasche-Orlow and Wolf (16). In this model, health literacy determines health outcomes via three main pathways: healthcare access and utilization, patient-provider interaction, and self-care behaviors. Applied to lung cancer, oral health literacy specifically affects how patients obtain and understand oral health information, communicate oral symptoms and care needs to providers, and manage daily oral self-care during antitumor therapy.

However, the extent to which patients successfully translate oral health literacy into effective self-care may depend on their psychosocial context. The empirical evidence reviewed above—particularly the mediation roles of self-efficacy and control efficacy in lung cancer patient (17), the direct associations of family support with psychological outcomes (13), and the mediating effect of oral health-related self-efficacy between oral health literacy and oral health outcomes (14)—supports the theoretical contention that psychological distress (e.g., anxiety), external support availability (e.g., family support), and perceived behavioral capability (e.g., self-efficacy) are three essential components that can either facilitate or hinder the conversion of oral health knowledge into sustained oral health behaviors. Accordingly, anxiety, family support, and self-efficacy were selected as core indicators of psychosocial factors in this study.

Based on the above literature review, the novelty and necessity of this study are threefold. First, although the relationship between oral health literacy and oral health has been validated in the general population, empirical evidence is lacking specifically for lung cancer patients—a population facing unique treatment-related oral complications (e.g., oral mucositis, xerostomia) and high psychological burden, where the OHL–oral health relationship may exhibit different patterns. Second, while psychosocial factors (anxiety, self-efficacy, and social support) have been separately shown to influence disease management behaviors in lung cancer patients and the relationship between oral health literacy and health outcomes, their integrated mediating role in the “OHL → oral health” pathway has not been systematically examined. Third, previous studies have predominantly focused on head and neck or oral primary tumor patients; lung cancer patients present a unique clinical context because the anatomical connection between the oral cavity and the lower respiratory tract means that poor oral health may directly trigger pulmonary infections—a serious complication that makes this investigation particularly clinically relevant.

Therefore, this study aims to establish a mediating effects model involving oral health literacy, psychosocial factors, and oral health status in lung cancer patients, thereby providing a basis for developing targeted intervention strategies and ensuring the smooth implementation of anticancer treatment.

## 2 Methods

### 2.1 Study design and setting

This study is a cross-sectional questionnaire survey targeting lung cancer patients receiving treatment at tertiary hospitals in China. The study aims to explore the intrinsic relationships among oral health literacy, psychosocial factors, and oral health status. This research report adheres to the Strengthening the Reporting of Observational Studies in Epidemiology (STROBE) (18).

### 2.2 Participants and sampling

This study employed convenience sampling to recruit lung cancer patients admitted to a tertiary hospital in China. The recruitment followed a two-phase design that was pre-specified in the original study protocol prior to data collection: Phase 1 (April–July 2025, *n* = 135) served as the initial enrollment period, and Phase 2 (April–June 2026, *n* = 115) was planned as an extension to achieve the target sample size (*N* = 250) required for structural equation modeling. All participants across both phases were recruited from the same tertiary hospital using identical eligibility criteria (see Inclusion and Exclusion criteria below) and standardized study procedures. No changes were made to the clinical setting, questionnaire battery, or data collection protocols between the two phases. The study protocol, including the planned two-phase recruitment and the final target sample size, was approved by the institutional ethics committee (approval number: 2025YS-067-01) prior to the commencement of Phase 1, and no protocol amendments were made regarding the recruitment design or sample size during the study period.

To evaluate whether the final sample size was adequate for the structural equation model, we conducted a parameter-based Monte Carlo power analysis with 5,000 replications. The data-generating model reproduced the final SEM, including two latent constructs measured by three indicators each, the direct and indirect paths from oral health literacy to oral health, and adjustment for age. Population parameter values and residual variances were taken from the model fitted to the complete sample (*N* = 250). For each replication, a sample of 250 observations was generated and the full SEM was re-estimated using maximum likelihood. Statistical power was defined as the proportion of converged replications in which the two-sided test was significant at *α* = 0.05. The indirect effect was evaluated as the product of the two component paths using a first-order delta-method standard error. Monte Carlo standard errors were reported to quantify simulation uncertainty. All 5,000 simulated datasets converged. Estimated power was 100.0% for the oral health literacy-to-psychosocial path, 98.5% for the psychosocial-to-oral health path, 99.9% for the adjusted direct effect of oral health literacy on oral health, and 98.2% for the indirect effect. The Monte Carlo standard error for the indirect-effect power estimate was 0.19 percentage points. These results indicate that *N* = 250 provided adequate power for the key structural and mediation effects under parameter values consistent with the fitted model.

Inclusion criteria were: (a) meeting the diagnostic criteria for lung cancer outlined in the *Clinical Diagnosis and Treatment Guidelines for Lung Cancer (2024 Edition)* (19) of the Chinese Medical Association; (b) age ≥ 18 years; (c) Normal communication ability; (d) Voluntary participation in the study. Exclusion criteria: (a) Concurrent malignant tumors in other systems; (b) Severe complications such as multi-organ failure; (c) Individuals with a history of or currently suffering from mental disorders, cognitive impairments, or dementia who are unable to read or complete the questionnaire.

The sample size estimation for this study considered both the cross-sectional survey design and the requirements of structural equation modeling (SEM). For the cross-sectional descriptive analysis, the sample size was determined based on the rule of 5–10 times the number of independent variables (21 variables), yielding a required sample of 117–234 cases accounting for a 10% attrition rate (20). For the SEM analysis, we adopted the widely recommended criterion of at least 10 cases per estimated parameter. Our hypothesized model included 21 estimated parameters (factor loadings, path coefficients, and error variances), requiring a minimum of 210 cases. Considering the cross-sectional nature and the SEM requirements, a total of 250 cases were ultimately enrolled in this study.

### 2.3 Measures

#### 2.3.1 Socio demographic and disease characteristics

The researchers developed the demographic questionnaire independently based on a systematic review and analysis of relevant literature. It includes 15 items: gender, age, educational level, employment status, marital status, place of residence, annual household income, type of health insurance, presence of a caregiver, smoking history, duration of illness, treatment modality, number of comorbid chronic conditions, dietary habits, and whether the participant wears dentures.

#### 2.3.2 Psychosocial factors

The grouping of anxiety, family support, and self-efficacy into a single psychosocial latent construct is grounded in Bandura’s Social Cognitive Theory and the stress-buffering model of social support. These frameworks collectively posit that psychosocial functioning is determined by the interplay of three core components: (1) psychological distress (emotional state), (2) external resources (social support), and (3) internal resources (self-efficacy beliefs). Specifically, anxiety represents the psychological distress component—a negative emotional state that can impair cognitive function, emotional regulation, and health behaviors; family support represents the external resource component—the availability of emotional and instrumental assistance from the family environment, which provides a buffer against stress and facilitates health-promoting behaviors; and self-efficacy represents the internal resource component—an individual‘s belief in their capability to execute behaviors necessary to achieve desired outcomes. These three components are theoretically and empirically interconnected: social support shapes self-efficacy through vicarious experience and verbal persuasion, and higher self-efficacy in turn reduces anxiety by enhancing perceived control over threatening situations. Consistent with this theoretical framework, we selected the Self-Rating Anxiety Scale (SAS) to measure psychological distress, the Perceived Social Support from Family scale (PSS-Fa) to measure external resources, and the Cancer Self-Efficacy Scale (C-SUPPH) to measure internal resources. These three indicators are expected to co-vary as manifestations of an individual’s overall psychosocial functioning, a premise empirically supported by studies demonstrating chain mediating effects among family support, self-efficacy, and anxiety (12, 21).

##### 2.3.2.1 Self-efficacy in self-management

Self-efficacy in self-management was measured using the Cancer Self-Efficacy Scale (C-SUPPH). This scale was translated into Chinese by Qian Huijuan et al. (22) in 2011. It comprises three dimensions—self-stress reduction (3 items), positive attitude (15 items), and self-decision-making (10 items)—for a total of 28 items. A higher score indicates higher self-efficacy among patients. Based on the scoring range: ≤65 points indicates low self-efficacy; 66–102 points indicates moderate self-efficacy; ≥103 points indicates high self-efficacy. The scale demonstrates good reliability and validity, with a Cronbach’s *α* coefficient of 0.970 and a test–retest reliability of 0.937.

##### 2.3.2.2 Anxiety

Anxiety was measured using the Self-Rating Anxiety Scale (SAS). This scale was developed by Zung (23) in 1971. It consists of 20 items, each rated on a 4-point scale. The cumulative scores of all items constitute the SAS total score, which is then multiplied by 1.25 and rounded to the nearest whole number to obtain the standard score. Scores are positively correlated with anxiety levels. The standard cutoff score is 50; scores <50 are considered normal, 50–59 indicate mild anxiety, 60–69 indicate moderate anxiety, and ≥70 indicate severe anxiety. The Cronbach’s *α* coefficient for this scale is 0.872, and its validity coefficient is 0.840. In this study, the raw score of this scale is reported in descriptive statistics (Table 1), while the reverse-coded score is used in all correlation and SEM analyses.

**Table 1:** Raw scores for psychosocial factors and oral health status (*N* = 250).

Variables	Mean (SD)
Anxiety	57.29 ± 8.29
Family support	8.37 ± 2.73
Self-efficacy	89.77 ± 11.91
Oral health status	9.14 ± 1.94
Oral health behaviors	2.76 ± 1.79
Oral health impact	38.28 ± 4.71
Oral Health Literacy	28.07 ± 5.39

*All scores are presented as raw scores in their original direction. For SAS, BOAS, and OHIP-14, higher raw scores indicate worse status.

##### 2.3.2.3 Family support

Family support was measured using the Perceived Social Support From Family scale (PSS-Fa). This scale, developed by Procidano et al. (24) in 1983, consists of 15 items. Items are answered with “Yes” or “No,” with “Yes” scoring 1 point and “No” scoring 0 points, resulting in a total score range of 0–15 points. A score of ≥10 indicates high family support, while a score of <10 indicates low family support. In this study, the Cronbach’s *α* coefficient for the scale was 0.803.

#### 2.3.3 Oral health literacy

Oral health literacy was assessed using the Short Form of Health Literacy Dental Scale (HeLD-14). This scale was translated into Chinese by Yan et al. (25) in 2021 to assess patients’ oral health literacy. It comprises 14 items across seven dimensions: acceptance, understanding, support, finances, healthcare-seeking, communication, and application. The total score ranges from 0 to 56 points, with higher scores indicating greater oral health literacy. The scale’s overall Cronbach’s alpha coefficient is 0.908, and its test–retest reliability is 0.988.

#### 2.3.4 Oral health

Following the same logic as for psychosocial factors, the three oral health indicators—BOAS, HBS, and OHIP-14—were specified as manifestations of a single latent construct. This approach is grounded in the FDI theoretical framework for oral health (26), which conceptualizes oral health as a multidimensional construct comprising clinical signs, behavioral practices, and patient-reported impacts. Accordingly, BOAS was selected to capture the clinical domain (objective signs of oral disease), HBS to capture the behavioral domain (oral hygiene practices), and OHIP-14 to capture the patient-reported impact domain (functional and psychosocial consequences of oral conditions). This multidimensional yet unified approach is consistent with contemporary conceptualizations of oral health as a latent construct that manifests through multiple observable indicators (27).

##### 2.3.4.1 Oral health status

Oral health status was assessed using the Beck Oral Assessment Score (BOAS). This scale was translated into Chinese by Pan et al. (28) in 2019 to assess patients’ oral health status. It includes five assessment items: lips, gums/oral mucosa, tongue, teeth, and secretions. Each item is scored on a 1–4 point scale, with a total score ranging from 5 to 20. A higher score indicates more severe oral disease and poorer oral health. A score of 5 indicates no impairment, 6–10 indicates mild impairment, 11–15 indicates moderate impairment, and 16–20 indicates severe impairment. The scale’s overall Cronbach’s *α* coefficient is 0.902. In this study, the raw score of this scale is reported in descriptive statistics (Table 1), while the reverse-coded score is used in all correlation and SEM analyses.

##### 2.3.4.2 Oral health behaviors

Oral health behaviors were measured using the Hygiene Behavior Scale (HBS). This scale was developed by Zhang et al. (29) in 2024 to assess patients’ oral health behaviors. It consists of 10 items with a maximum score of 10 points; a total score of ≥8 indicates good oral hygiene, while a score of <8 indicates poor oral hygiene. The scale’s Cronbach’s *α* is 0.804.

##### 2.3.4.3 Oral health impact

Oral health impact was measured using the Oral Health Impact Profile-14 (OHIP-14). This scale was translated into Chinese by Xin et al. (30) in 2006 to assess patients’ oral health-related quality of life. It consists of four dimensions and 14 items: physical discomfort (3 items), pain and discomfort (3 items), psychological distress (3 items), and reduced independence (5 items). All items use a 5-point Likert scale, with scores ranging from 0 (never) to 4 (often). The total score ranges from 0 to 56, with higher scores indicating poorer oral health-related quality of life. The scale’s overall Cronbach’s alpha coefficient is 0.930. In this study, the raw score of this scale is reported in descriptive statistics (Table 1), while the reverse-coded score is used in all correlation and SEM analyses.

### 2.4 Data collections procedure

**Figure 1:**
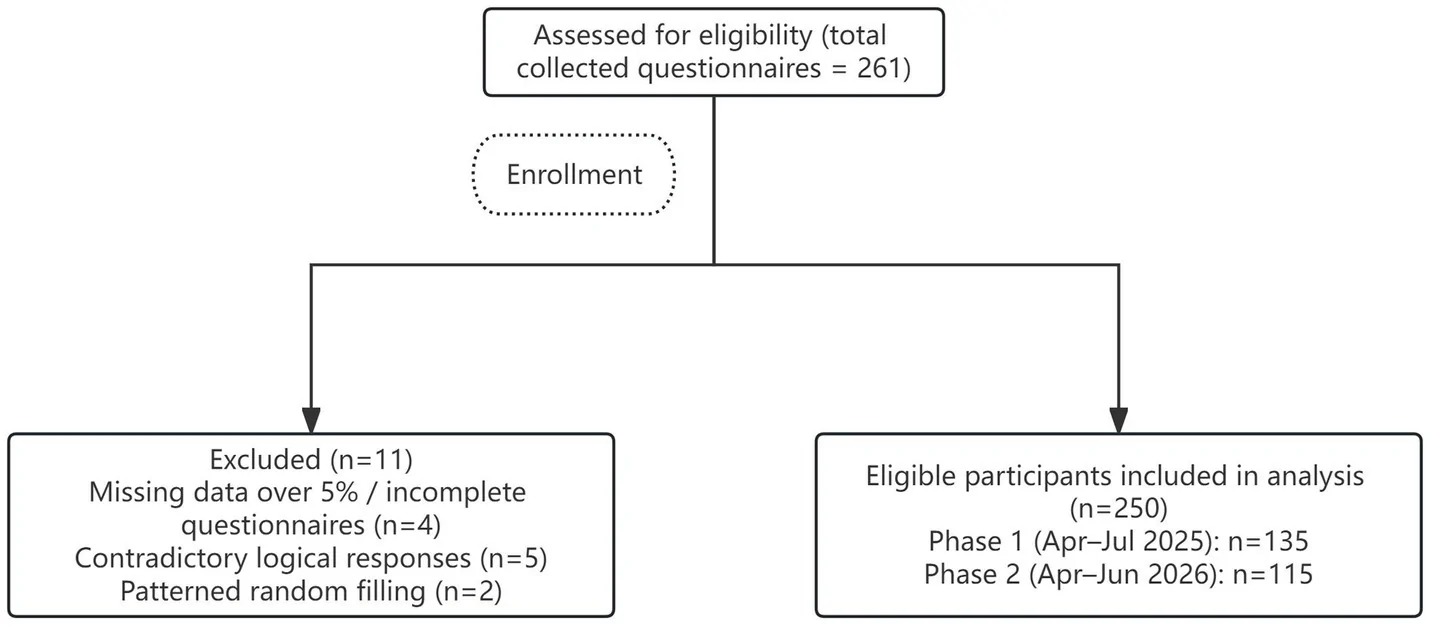
Participant flow diagram.

Research team members underwent standardized training to ensure consistent explanations of questionnaire items to patients. After the questionnaires were returned, they were checked item by item; if any items were missing, patients were reminded to complete them. Data was organized and entered by two independent staff members, and questionnaires with obvious logical errors were excluded. The recruitment process was carried out in two phases: an initial phase from April to July 2025 (*n* = 135) and an expansion phase from April to June 2026 (*n* = 115). A total of 261 questionnaires were collected across both phases, yielding 250 valid responses (135 from the initial phase and 115 from the expansion phase), for a valid response rate of 95.78%. The average time taken to complete the questionnaire was (21.2 ± 3.2) minutes. The participant flow diagram (Figure 1) clearly presents the number of screened, eligible, enrolled, excluded and analyzed participants at each stage. For cases with isolated missing items (less than 5% of cases, with missingness completely at random), multiple imputation using the fully conditional specification method in SPSS (version 26.0) with 5 imputation iterations was applied to handle missing responses prior to analysis. Questionnaires with obvious logical errors (e.g., contradictory responses or patterned responding) were excluded from the analysis. Prior to conducting the formal statistical analyses, a rigorous data verification process was undertaken to ensure the accuracy of the expanded dataset. Re-examination of the raw questionnaire data identified two issues: (1) incorrect application of reverse-coding for negatively framed scales (SAS, BOAS, and OHIP-14), where raw scores were mistakenly entered without the required transformation; and (2) minor variable misalignment in the initial coding sheet for a subset of cases. To rectify these issues, two independent research assistants re-entered all raw data from the original paper questionnaires and performed a side-by-side comparison to resolve any discrepancies. All reverse-coded items were then cross-checked against the original response sheets to ensure accurate transformation (e.g., 25 − BOAS_raw, 56 − OHIP-14_raw, and 100 − SAS_raw). Finally, logical consistency checks and recalculation of bivariate correlations confirmed that all associations aligned with the theoretically expected directions.

### 2.5 Statistical analysis

This study used SPSS 26.0 for descriptive and statistical analysis. Continuous variables were described as mean ± standard deviation, while categorical variables were described as frequency and percentage.

Prior to all correlation and structural equation modeling analyses, all variables were uniformly coded such that higher scores indicated better oral health status or more favorable psychosocial conditions. Specifically, SAS, BOAS, and OHIP-14 scores were reverse-coded using the formula: recoded score = maximum possible score − raw score (i.e., 25 − BOAS_raw, 56 − OHIP-14_raw, and 100 − SAS_raw). HBS, HeLD-14, PSS-Fa, and C-SUPPH were retained in their original scoring directions, as higher scores already indicate better status. This standardization ensures that the interpretation of correlation coefficients and path estimates is consistent across constructs. For descriptive statistics (Table 1) and clinical interpretation, the raw scores of the three negatively framed scales—SAS, BOAS, and OHIP-14—are presented in their original direction, where higher scores indicate worse status. For all inferential analyses (Pearson correlation and structural equation modeling), these scores were reverse-coded using the formulas: 100 − SAS_raw, 25 − BOAS_raw, and 56 − OHIP-14_raw, respectively, so that higher scores uniformly indicate better status, consistent with the coding of the other variables. This distinction is clearly noted in the relevant sections and table footnotes.

To examine interrelationships among participants’ psychosocial factors, oral health status, and oral health literacy, Pearson’s correlation analysis was conducted.

Prior to testing the structural model, we employed a two-step analytic approach to establish the measurement properties of the composite constructs. First, we conducted exploratory factor analysis (EFA) for each composite construct (oral health status and psychosocial factors). The purpose of the EFA was not to discover an unknown factor structure, but rather to empirically validate the unidimensionality of the theoretically specified composite constructs—specifically, to confirm that the selected indicators, although derived from different measurement instruments with different scoring metrics, converged onto a single latent factor as expected. This step is particularly important when combining indicators from different scales into a composite construct, as it provides empirical assurance that the aggregation does not introduce unintended multidimensionality. Given that each construct comprised only three indicators, the EFA served as a preliminary data-driven check of construct coherence. Following the EFA, we employed confirmatory factor analysis (CFA) within the structural equation modeling framework to formally test the measurement model, including assessments of composite reliability (CR), average variance extracted (AVE), and discriminant validity. To establish the distinctiveness of the three latent constructs—oral health literacy, psychosocial factors, and oral health status—we assessed discriminant validity using the Fornell-Larcker criterion. The square root of the average variance extracted (AVE) for each construct was compared with the correlations between constructs. This two-step strategy is consistent with recommended practices in health behavior research when combining multiple indicators into latent constructs.

**Figure 2:**
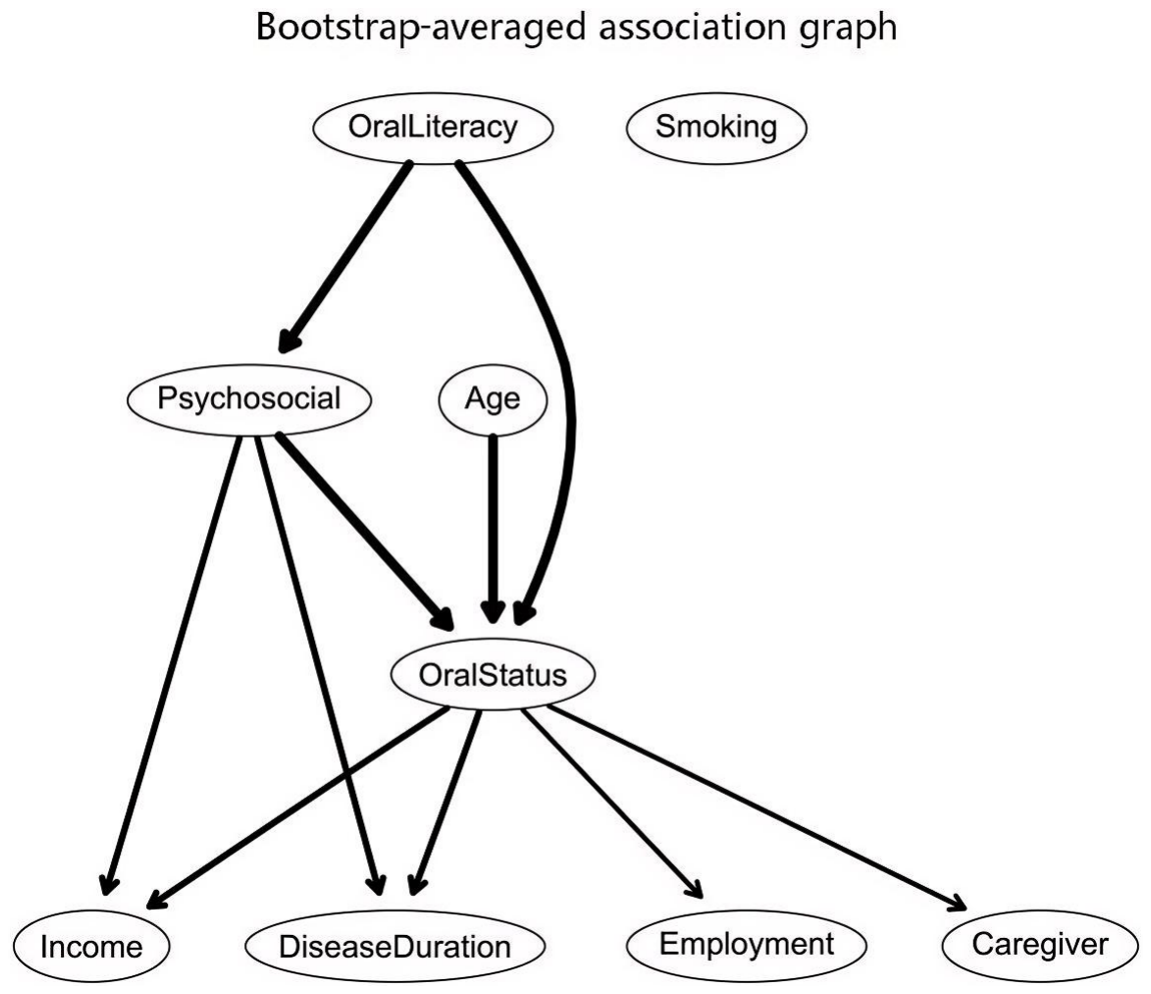
Bootstrap-averaged data-driven association graph presenting statistically dominant statistical association patterns across 5,000 resampling iterations. Ellipses represent latent constructs, and solid arrows denote statistically recurrent directional associations detected in bootstrap sampling. OHL = oral health literacy; Psychosocial = latent composite of anxiety, family support, and self-efficacy; OralStatus = latent composite of oral health clinical status, oral hygiene behaviors, and oral health-related quality of life impact; Income = annual household income; DiseaseDuration = self-reported disease course; Employment = post-diagnosis employment status; Caregiver = family caregiver availability. This data-driven association graph identified age as the sole direct confounder that requires statistical adjustment when estimating the total effect of oral health literacy on oral health status.

To guide covariate adjustment in the structural equation model, we first identified a set of candidate confounders based on prior clinical knowledge and a systematic literature review (16, 31, 32), including age, smoking history, annual household income, disease duration, employment status, and caregiver availability—all of which have been previously reported to influence oral health or health literacy in cancer populations. To empirically determine which of these candidate variables should be adjusted for as confounders in our specific sample, we conducted a purely exploratory data-driven graphical analysis using bootstrap resampling (5,000 iterations). It is important to emphasize that this exploratory graph is entirely data-derived from our cross-sectional sample and is not theory-driven; it does not represent a conventional causal Directed Acyclic Graph (DAG), nor does it imply causal or temporal sequences. The directional arrows in Figure 2 are purely statistical outputs derived from bootstrap averaging of cross-sectional data and should be interpreted only as empirical association patterns.

Furthermore, AMOS 26.0 software was used to construct the structural equation model. Model fit was then evaluated using the chi-square-to-degrees-of-freedom ratio (*χ*^2^/df), the Comparative Fit Index (CFI), the Tucker-Lewis Index (TLI), the Root Mean Square Error of Approximation (RMSEA), and the Standardized Residual Mean Square Root (SRMR). The fit criteria were as follows: *χ*^2^/df < 3 (52), CFI and TLI ≥ 0.90 (53), RMSEA < 0.08 (54), and SRMR < 0.08 (55).

Additionally, the Bootstrap method was used to test the mediating effect of psychosocial factors on the relationship between oral health literacy and oral health status. Given the cross-sectional nature of the data, the mediation analysis was performed as an exploratory assessment of indirect associations, consistent with common practices in health behavior research. The Bootstrap results should be interpreted as providing evidence of statistical compatibility with the proposed pathway, rather than as establishing temporal causality. The significance level was set at *α* = 0.05.

### 2.6 Ethical considerations

This study has been approved by the ethics committee of the participating hospital (approval number: 2025YS-067-01). Before the survey began, the purpose and content of the study were explained to the patients to ensure informed consent and voluntary participation. During the study, questionnaires were recorded using codes to protect patient privacy. The survey process complies with ethical principles governing research involving human subjects, with patient safety as the primary consideration.

## 3 Results

### 3.1 Demographic characteristics, psychosocial factors, and oral health status of lung cancer patients

This study included 250 lung cancer patients. Among them, 74.8% were male, and 25.2% were female; the majority were aged 60 years or older (60.4%); in terms of educational attainment, 56.8% had completed junior high school or less; the majority were unemployed (82.4%); and slightly more were enrolled in urban medical insurance (53.2%). Regarding disease and treatment-related characteristics, 40.8% had a smoking history longer than 20 years. Over half (52.4%) had a disease duration of less than 1 year, while 56.0% had two or fewer comorbidities. Most received single-agent chemotherapy (58.4%), primarily ate regular food (93.6%), and had dentures (71.6%) (See Table 2). The oral health literacy score was (28.07 ± 5.39) points (See Table 1). All descriptive statistics are presented in Table 1 using raw scores.

**Table 2:** Demographic data on lung cancer patients (*N* = 250).

Variable	*N*	%
Gender		
Male	187	74.8%
Female	63	25.2%
Age		
<45	18	7.2%
45 ~ <60	81	32.4%
≥60	151	60.4%
Educational level		
Elementary school and below	47	18.8%
Junior high school	95	38.0%
High school/vocational high school/technical secondary school	82	32.8%
College and above	26	10.4%
Employment status		
Employed	44	17.6%
Unemployed	206	82.4%
Marital status		
Unmarried	11	4.4%
Married	149	59.6%
Divorced	28	11.2%
Widowed	62	24.8%
Place of residence		
City/Town	132	52.8%
Rural	118	47.2%
Annual household income		
<30,000	66	26.4%
30,000 ~ <50,000	63	25.2%
50,000 ~ <100,000	59	23.6%
≥100,000	62	24.8%
Health insurance type		
Urban employee/resident medical insurance	133	53.2%
New rural cooperative medical scheme	117	46.8%
Caregiver		
Attendant	32	12.8%
Spouse	88	35.2%
Children	78	31.2%
Parents	27	10.8%
Other relatives and friends	25	10.0%
Smoking history		
≤5 years	53	21.2%
5 years~ ≤ 10 years	46	18.4%
10 years~ ≤ 20 years	49	19.6%
>20 years	102	40.8%
Disease duration		
≤1 year	131	52.4%
1 year~ ≤ 3 years	53	21.2%
3 year~ ≤ 5 years	35	14.0%
>5 years	31	12.4%
Treatment methods		
Chemotherapy	146	58.4%
Surgery + Chemotherapy	104	41.6%
Number of chronic conditions		
≤2	140	56.0%
3 ~ 4	69	27.6%
≥5	41	16.4%
Dietary type		
General diet	234	93.6%
Diabetic diet	16	6.40%
With or without dentures		
Yes	179	71.6%
No	71	28.4%

### 3.2 Exploratory factor analysis of oral health and psychosocial factors in lung cancer patients

Exploratory analysis results showed a KMO value of 0.710 and a significant Bartlett’s sphericity test (*χ*^2^ = 286.095, *df* = 3, *p* < 0.001), indicating that the scale is suitable for factor analysis. Principal component analysis identified a single factor, which accounted for 74.60% of the total variance. Accordingly, this common factor was named “Oral Health Status”(See Table 3).

**Table 3:** Results of an exploratory factor analysis of oral health status.

Oral health	Factor load
BOAS	0.871
HBS	0.888
OHIP-14	0.832

Exploratory analysis results showed a KMO value of 0.749 and a significant Bartlett’s sphericity test (*χ*^2^ = 435.425, *df* = 3, *p* < 0.001), indicating that the scale is suitable for factor analysis. Principal component analysis yielded a single factor, accounting for 82.38% of the total variance. Accordingly, this common factor was named “Psychosocial Factors”(See Table 4).

**Table 4:** Results of exploratory factor analysis of psychosocial factors.

Psychosocial factors	Factor load
Anxiety	0.914
Family support	0.901
Self-efficacy	0.908

Despite the limited number of indicators (three per construct), the EFA yielded clear and interpretable results, with all factor loadings exceeding 0.83 and each single-factor solution accounting for a substantial proportion of the total variance (74.60% for oral health status and 82.38% for psychosocial factors). These findings confirm that, despite originating from different measurement instruments, the indicators within each construct share sufficient common variance to justify their treatment as manifestations of a single latent factor.

### 3.3 Correlations and discriminant validity

Prior to testing the structural model, a two-step approach was employed, beginning with the assessment of the measurement model for the three latent constructs: oral health literacy, psychosocial factors, and oral health status. Confirmatory factor analysis (CFA) was conducted to evaluate the psychometric properties of these constructs. The CFA results, presented in Table 5, demonstrated acceptable factor loadings for all items (ranging from 0.694 to 0.888), indicating satisfactory indicator reliability. Composite reliability (CR) values for Oral Health Literacy (0.831) and Psychosocial Factors (0.872) exceeded 0.80; for Oral Health, CR was slightly below this threshold (0.762), which is acceptable given the limited number of indicators (three per construct) and the exploratory nature of this composite construct in the current sample. Convergent validity was supported by average variance extracted (AVE) values exceeding the recommended threshold of 0.50 for all constructs (ranging from 0.712 to 0.747). Discriminant validity was assessed using the Fornell–Larcker criterion; as shown in the lower portion of Table 5, the square root of the AVE for each construct (ranging from 0.844 to 0.864) was greater than its correlations with the other constructs (ranging from 0.333 to 0.402), confirming that the three constructs are distinct.

**Table 5:** Discriminant validity and inter-construct correlations among study variables (*N* = 250).

Variables	Factor loadings range	CR	AVE	√AVE	1	2	3
1. Oral health literacy	0.702–0.873	0.831	0.712	0.844	1.000	–	–
2. Psychosocial factors	0.842–0.875	0.872	0.747	0.864	0.333^*^	1.000	–
3. Oral health	0.694–0.888	0.762	0.741	0.861	0.389^*^	0.402^*^	1.000

CR, Composite Reliability; AVE, Average Variance Extracted; √AVE, square root of AVE (bolded on diagonal). All variables were coded such that higher scores indicate better oral health status or more favorable psychosocial conditions. BOAS, OHIP-14, and SAS scores were reverse-coded prior to analysis. *p < 0.001 (two-tailed).

The Pearson correlation matrix is also presented in Table 5. Oral health status was significantly positively correlated with oral health literacy (*r* = 0.389, *p* < 0.001) and psychosocial factors (*r* = 0.402, *p* < 0.001); oral health literacy was also significantly positively correlated with psychosocial factors (*r* = 0.333, *p* < 0.001).

### 3.4 Exploratory data-driven association graph analysis

The data-driven bootstrap-averaged association graph (Figure 2) illustrated the statistically dominant statistical association patterns extracted from bootstrap resampling, with prior clinical literature only used to constrain variable inclusion scope rather than fix directional paths in advance. This graph was not pre-specified by pure theoretical reasoning; instead, we conducted 5,000 bootstrap resampling iterations on the cross-sectional dataset, generated a unique association graph for each resample, then averaged all valid association graph structures to retain only the most frequently detected directional arrows across all bootstrap samples, forming the final visualized data-driven graph. Oral health literacy (OHL) was identified as the most distal upstream predictor by bootstrap averaging, with direct paths to both psychosocial factors and oral health status. Psychosocial factors served as a key mediating hub, receiving direct input from OHL and, in turn, directly affecting oral health status. Oral health status was statistically identified as directly influenced by OHL (direct path), psychosocial factors, and age. Critically, the association graph identified the minimally sufficient adjustment set for estimating the total effect of OHL on oral health status: only age met the criteria for direct confounding. Smoking history, income, disease duration, employment status, and caregiver availability were not identified as direct confounders of the OHL–oral health relationship; rather, bootstrap averaging consistently yielded arrows pointing from oral health status to these four variables, which appears counterintuitive to conventional population-level temporal ordering, and we supplement the post-hoc directional interpretation of this data-derived directional pattern below. Based on these association graph findings, age was selected as the sole covariate for adjustment in the subsequent structural equation model.

### 3.5 Testing the hypothesized mediating pathway of psychosocial factors

**Figure 3:**
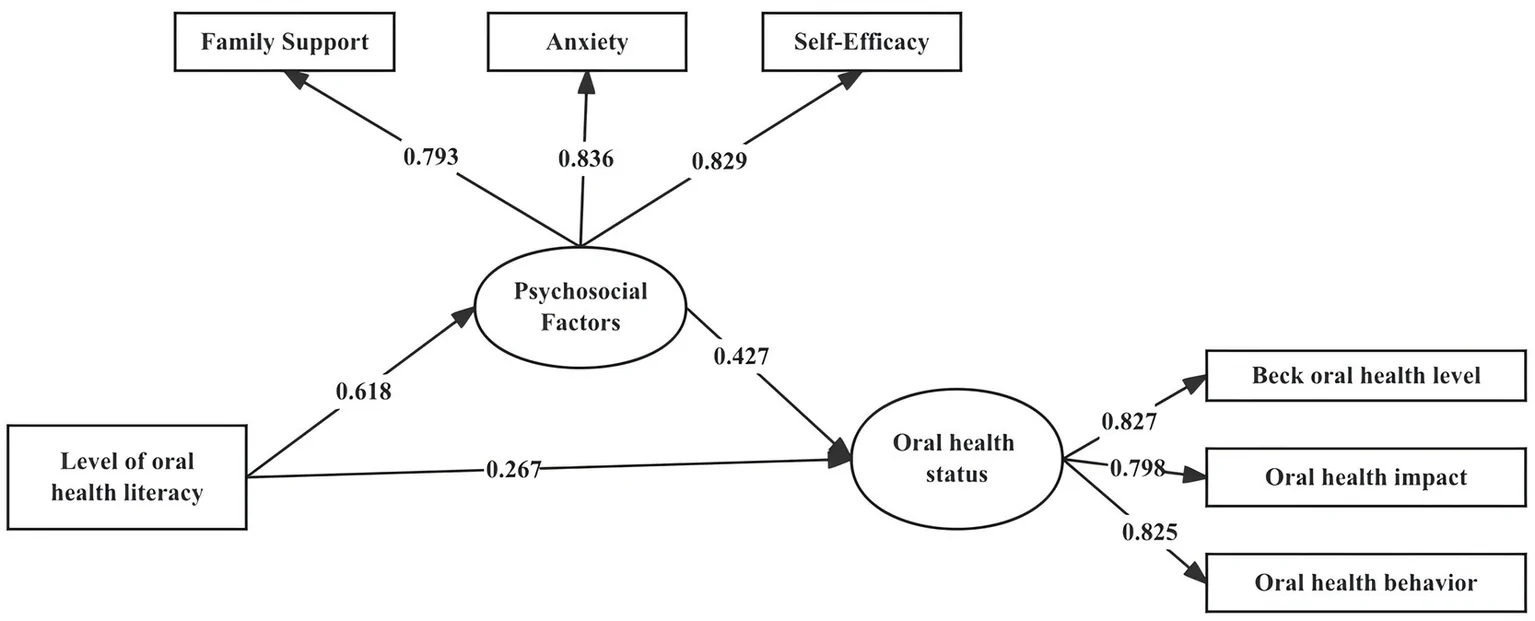
Diagram of the equation model for oral health status in lung cancer patients.

A structural equation model was constructed with oral health status as the dependent variable, oral health literacy as the independent variable, and psychosocial factors as the mediating variable (see Figure 3). The structural equation model demonstrated acceptable fit: *χ*^2^/df = 1.087, CFI = 0.937, TLI = 0.929, RMSEA = 0.019, SRMR = 0.046. All indices met the recommended thresholds, supporting the validity of the mediation analysis. The Bootstrap procedure was set to 5,000 iterations, and the criterion for validity was that the 95% confidence interval did not include zero. The results showed that all paths were statistically significant. The results are consistent with the hypothesized partial mediation model, suggesting that psychosocial factors may serve as an intermediary pathway linking OHL to oral health status in this cross-sectional sample. However, these findings remain exploratory and do not imply a causal sequence (See Table 6).

**Table 6:** Testing the mediating effects of psychosocial factors on the relationship between oral health status and oral health literacy among lung cancer patients.

Path	Effect size	95%CI	*p*	Proportion of effect (%)
Indirect effect	0.264	0.106–0.351	<0.001	49.7
Direct effect	0.267	0.119–0.378	<0.001	50.3
Total effect	0.531	0.439–0.718	<0.001	100.00

Estimates are adjusted for age as a covariate based on the minimally sufficient adjustment set identified by the association graph (Figure 2).

## 4 Discussion

This study demonstrates that lung cancer patients generally experience mild oral health impairment, as reflected in their BOAS raw score (9.14 ± 1.94; scores 6–10 indicate mild impairment per the scale’s original criteria), which aligns with previous findings by Chao Sheng et al. (31). Poor oral health, such as frequent gum bleeding, is relatively common among lung cancer patients. This may be related to complications commonly associated with cancer treatment, such as dry mouth, oral mucositis, and difficulty opening the mouth (33). However, this study found that lung cancer patients exhibit poor oral health behaviors, such as infrequent toothbrushing, and certain oral bacteria can form biofilms and produce acids under poor hygiene conditions, directly promoting the development of dental caries and exacerbating oral health problems (34). Furthermore, the poor oral health status defined as “rarely brushing teeth and always experiencing gum bleeding” was significantly associated with an increased risk of mortality in lung cancer patients (OR = 1.10; 95% CI = 1.05–1.16) (35). Furthermore, the high raw scores on the OHIP-14 (38.28 ± 4.71, where higher scores indicate greater negative impact) indicate poor oral health-related quality of life, which may lead to adverse outcomes such as frailty, hospitalization, and falls (36). Therefore, healthcare professionals should prioritize assessing oral health-related quality of life in lung cancer patients, identify high-risk individuals early, and strengthen guidance on oral hygiene behaviors and management of complications to reduce the risk of adverse outcomes.

Beyond the objective clinical signs reflected by the BOAS, a particularly striking finding of this study is the extremely low oral health behavior score (HBS = 2.76 ± 1.79, out of a maximum of 10 points), which is far below the threshold of ≥8 indicating good oral hygiene. This score suggests that most patients engage in toothbrushing less than once daily or perform minimal oral hygiene practices. The marked discrepancy between the relatively mild objective oral impairment (BOAS 9.14 ± 1.94) and the profoundly poor self-reported behaviors warrants careful clinical attention. Several interconnected factors may explain this phenomenon. First, anticancer treatment-related fatigue, oral pain, and xerostomia can physically diminish patients’ ability and willingness to perform routine toothbrushing, making even simple self-care tasks burdensome. Second, the overwhelming psychological burden of a lung cancer diagnosis, coupled with intensive treatment schedules, may substantially lower the perceived priority of oral hygiene relative to life-saving antitumor therapy, leading to its neglect. Third, chemotherapy-induced cognitive decline or depressive symptoms may impair executive function and reduce motivation for daily self-care behaviors. From a clinical perspective, this behavioral deficit carries significant implications. Given the anatomical continuity between the oral cavity and the lower respiratory tract, poor oral hygiene directly facilitates the colonization and aspiration of periodontal pathogens, thereby increasing the risk of aspiration pneumonia—a potentially life-threatening complication in lung cancer patients already burdened by compromised pulmonary reserve. Therefore, healthcare providers should not assume that patients’ objective oral status reflects their actual daily hygiene practices. Instead, routine clinical assessment should incorporate direct inquiries about toothbrushing frequency and techniques, coupled with the provision of simplified, adaptive oral care protocols (e.g., soft-bristled brushes, alcohol-free mouth rinses, and family-assisted hygiene) integrated into standard oncology nursing pathways.

Oral health literacy in lung cancer patients is notably low, as shown by this study’s overall score rate of 50.13%. This level is not only significantly lower than that of the general dental patient population (37, 38) but may even be lower than that of the general older adult(s) population (39). Possible reasons for this include the psychological stress caused by the diagnosis of lung cancer and intensive anticancer treatments (40, 41). Consequently, lung cancer patients’ energy and focus are highly concentrated on the task of fighting the disease, leading to the neglect of oral health—an issue that may seem “secondary” but actually impacts quality of life. In terms of dimension scores, the results of this study indicate that the “understanding” dimension scored the highest, while the “seeking care” dimension scored the lowest. This reveals a clear disconnect between knowledge and action regarding oral health literacy among lung cancer patients. On the one hand, due to their long-term illness, these patients possess a certain level of medical literacy, enabling them to read and understand information related to oral health (42); on the other hand, since the sample population was drawn from a cardiopulmonary specialty hospital, factors such as referral to dental specialty hospitals and travel distance act as barriers to oral healthcare access for lung cancer patients (43). This suggests that healthcare professionals should adopt a holistic perspective on multimorbidity in chronic disease management and promptly provide patients with comprehensive, systematic care pathways that adapt to changes in oral health risks.

The latent variable approach adopted in this study is theoretically grounded in established frameworks—the FDI multidimensional oral health framework for the oral health construct and Social Cognitive Theory with the stress-buffering model for the psychosocial construct. The empirical support from our factor analyses (high loadings and satisfactory discriminant validity) confirms that these theoretical specifications are appropriate for our sample, reinforcing the validity of the subsequent mediation analysis. However, it is important to clarify the intended use of this approach: the latent variable “Oral Health Status” is primarily a research tool for testing theoretical relationships in structural equation modeling, not a clinical assessment instrument for individual patient evaluation. While the construct demonstrates satisfactory psychometric properties, its composite nature---merging clinical signs (BOAS), behavioral practices (HBS), and patient-reported impacts (OHIP-14)––means that a given total score could arise from multiple different clinical scenarios. For instance, two patients with identical latent variable scores might have entirely different profiles: one might exhibit severe oral mucosal lesions but maintain good hygiene practices and report low functional impact, while another might show mild clinical signs but report profound psychological distress due to oral health problems. From a clinical management perspective, these two patients would require fundamentally different interventions, yet their composite scores would suggest similar overall oral health status. Therefore, we emphasize that this latent variable is primarily a research tool for testing theoretical pathways in structural equation modeling, not a substitute for the clinical use of the original validated instruments. Clinicians should continue to interpret BOAS, HBS, and OHIP-14 according to their established scoring guidelines and clinical thresholds for specific decision-making. The composite score may serve as a supplemental screening indicator for overall oral health risk stratification, but its practical utility in routine clinical workflows requires further validation in prospective implementation studies.

Oral health literacy is positively associated with oral health status among lung cancer patients, as demonstrated in this study. The higher the level of oral health literacy, the better the oral health status. A cross-sectional study conducted in France from 2023 to 2024 explicitly reported that oral health literacy is positively correlated with oral health knowledge (*p* < 0.001) (7). This implies that individuals with higher oral health literacy are more likely to possess accurate oral health knowledge, better understand the importance of oral health, and be aware of methods for the prevention, treatment, and care of oral diseases, thereby adopting appropriate preventive measures (44). Given that older adults constitute a significant proportion of lung cancer patients (32), poor oral health in this population can have serious implications for their quality of life, self-esteem, and nutritional status (45). Therefore, this suggests that healthcare professionals should incorporate the assessment of oral health literacy and basic health education for lung cancer patients into routine care, and encourage patients to strengthen their oral health management.

As shown in the data-driven bootstrap-averaged association graph (Figure 2), several arrows run from oral health status to income, disease duration, employment status and caregiver availability, which conflicts with conventional population temporal sequences. It is critical to emphasize that all directional paths in Figure 2 are purely statistical outputs generated by averaging 5,000 bootstrap samples, rather than theoretically pre-specified causal structures. We provide post-hoc clinical reasoning to reconcile this data-derived pattern with the real clinical context of lung cancer cross-sectional samples: ① Disease duration: All participants were enrolled after confirmed lung cancer diagnosis, so objective disease onset chronologically precedes oral health assessment. However, our questionnaire collected subjective self-reported disease duration rather than objective diagnosis dates. Severe oral complications such as oral mucositis and xerostomia aggravate physical discomfort and symptom burden, leading patients to subjectively overestimate their disease course. This feedback association was repeatedly detected across bootstrap iterations, forming the directional arrow from oral health status to self-reported disease duration. ② Income and employment status: The cross-sectional survey only captured patients’ occupational and economic conditions after anti-tumor treatment, without collecting pre-diagnosis baseline data. Functional impairments caused by oral lesions reduce physical stamina and labor capacity, forcing patients to resign or cut working hours, which sequentially lowers household income. This post-diagnosis directional linkage was the dominant statistical signal in bootstrap averaging. ③ Caregiver: Although family support resources exist before cancer diagnosis, sustained full-time caregiver attendance only emerges when patients suffer severe oral dysfunction and lose self-care ability during treatment. The statistical correlation between poor oral health and increased caregiver reliance was consistently recovered across bootstrap samples, resulting in the downstream arrow of caregiver availability. From the perspective of directional inference, these four variables were excluded from the covariate adjustment set in the structural equation model for two core reasons. First, the data-driven association graph never identified direct pathways from income, disease duration, employment or caregiver availability to oral health literacy (the core exposure variable), which means they cannot satisfy the definition of pre-exposure confounders. Second, adjusting for these downstream statistical outcomes would introduce severe overadjustment bias, block the indirect effects of oral health literacy on long-term patient wellbeing, and distort the estimation of the mediating effect of psychosocial factors. We retained the full data-averaged arrow structure in Figure 2 to transparently report all statistical associations detected within our single-center sample, while the above theoretical interpretation helps clinical readers understand the clinical meaning behind these counterintuitive statistical paths.

According to the results of the mediation analysis in this study, within the hypothesised pathway, there is a significant indirect association between psychosocial factors and oral health literacy and oral health status among lung cancer patients. Psychosocial factors accounted for 49.70% of the total effect, suggesting that OHL may be directly associated with oral health status, and may also exert an indirect association through psychosocial factors. Since lung cancer patients often endure stress resulting from anticancer treatments, they are prone to developing adverse psychological states. A large-scale study from the UK Biobank (46) found that oral diseases frequently occur in patients with depression, anxiety, or comorbidities of both, which may be associated with elevated levels of inflammatory markers such as serum C-reactive protein and white blood cells. Furthermore, poor psychological states can impair an individual’s cognitive abilities, emotional regulation, and behavioral performance (47), which in turn affects patients’ ability to engage in self-care behaviors such as toothbrushing (48), thereby increasing the risk of tooth loss and the incidence of oral health diseases (49). This suggests that, in addition to routine treatment and care, healthcare professionals may consider assessing and monitoring lung cancer patients’ psychological well-being, strengthening collaboration with mental health counselors, and developing individualized psychological support plans—measures that may contribute to improved oral health outcomes, although longitudinal studies are needed to confirm this pathway. Social support can increase a sense of belonging, improve psychological well-being, enhance coping mechanisms, and alleviate symptom burden, thereby stimulating positive emotions and promoting mental health (50). The greater the perceived social support among lung cancer patients, the better equipped they are to cope with oral health issues (51). Since the primary sources of social support for lung cancer patients during hospitalization are healthcare providers and family members, this underscores the need for healthcare professionals to prioritize patients’ needs and provide both emotional and medical support. Additionally, healthcare providers should highlight the crucial role of family support and encourage patients to actively express their concerns, thereby gaining a better understanding of their needs. As noted above, psychosocial factors serve as a key mediating pathway through which oral health literacy influences the oral health status of lung cancer patients. Clinically, multidimensional collaborative intervention strategies should be developed to help improve patients’ oral health outcomes.

One additional methodological consideration deserves mention. The OHIP-14 includes a psychological discomfort dimension that conceptually overlaps with the psychosocial mediator (particularly anxiety), which may have partially inflated the observed indirect effect through shared method variance and construct overlap. Although our statistical analyses (including CFA and discriminant validity testing) confirmed that the latent constructs are empirically distinguishable, complete conceptual separation between oral health-related psychological impacts and general psychosocial functioning is difficult to achieve in questionnaire-based research. Therefore, the mediation effect size should be interpreted with caution, and future studies incorporating objective clinical oral health measures would help disentangle the unique contributions of these interrelated constructs.

## 5 Conclusion

The overall level of oral health literacy among lung cancer patients is relatively low, and psychosocial factors partially mediate the relationship between oral health literacy and oral health status. Therefore, improving the oral health status of lung cancer patients requires a dual focus on enhancing oral health literacy and leveraging psychosocial factors.

At the policy level, it is recommended that oral health care be incorporated into the primary health care system for cancer patients, and that financial investment in the management of cancer comorbidities be increased. At the clinical level, the association between cancer and oral diseases should be emphasized, and green channels for intra-hospital and inter-hospital oral care should be established to streamline the registration process. At the healthcare provider level, healthcare professionals should promptly assess patients’ oral health literacy and psychosocial factors, provide targeted oral health education and psychological support, and collaborate with family members to implement multidimensional intervention strategies to reduce the risk of oral complications and pulmonary infections, thereby improving patient outcomes.

Several limitations warrant consideration. First, this was a single-center cross-sectional study; all variables were measured concurrently, precluding causal inference despite the association graph-guided analytical framework. Second, while we used an exploratory association graph-informed approach (with variable selection guided by clinical literature but directional patterns determined empirically via bootstrap averaging) to systematically identify potential confounders and adjusted for age as the minimally sufficient set, residual confounding remains possible. The association graph suggested that variables such as income, disease duration, employment status, and caregiver availability operate primarily as mediators or downstream outcomes rather than as direct confounders; however, unmeasured or imperfectly measured factors—including detailed socioeconomic position, baseline oral hygiene habits, specific chemotherapy regimens, and cumulative treatment toxicity—could still contribute to residual confounding. Regarding smoking, although it appeared as an isolated node in our sensitivity analysis, this should not be interpreted as evidence against its biological relevance. Rather, this finding likely reflects the limited statistical power of our single-center sample, the relatively homogeneous distribution of smoking histories among patients receiving active anticancer treatment, or imprecise measurement of smoking exposure (e.g., pack-years vs. current/former status). Third, our sample size (*N* = 250) was adequate for the estimated parameters in the SEM, but constrained our ability to include all candidate covariates simultaneously without risking overfitting. Fourth, self-report measures may be influenced by social desirability, recall bias, and current mood states, which could inflate or deflate the observed associations. This limitation is inherent to the cross-sectional, questionnaire-based design and cannot be fully eliminated through statistical procedures alone. Future large-scale, multicenter prospective studies with comprehensive covariate measurement are needed to validate the directionality and robustness of the observed mediating pathway. Additionally, future research would benefit from incorporating objective clinical assessments of oral health (e.g., periodontal examinations, salivary biomarkers, or dental records) and observational measures of health behaviors, alongside self-reported data, to provide a more comprehensive and robust evaluation. Longitudinal designs that assess variables at multiple time points would also help to disentangle the directionality of effects and further reduce shared method variance.

## Data Availability

The datasets presented in this article are not readily available because access is restricted to protect patient privacy and confidentiality. Requests to access the datasets should be directed to the corresponding author, Tiane Fa, who will review requests on a case-by-case basis in accordance with ethical and institutional requirements.
